# Species interactions in three *Lemnaceae* species growing along a gradient of zinc pollution

**DOI:** 10.1002/ece3.8646

**Published:** 2022-02-18

**Authors:** Lorena Lanthemann, Sofia J. van Moorsel

**Affiliations:** ^1^ 27217 Department of Evolutionary Biology and Environmental Studies University of Zurich Zurich Switzerland

**Keywords:** Lemnaceae, species interactions, tolerance, zinc

## Abstract

Duckweeds (*Lemnaceae*) are increasingly studied for their potential for phytoremediation of heavy‐metal polluted water bodies. A prerequisite for metal removal, however, is the tolerance of the organism to the pollutant, e.g., the metal zinc (Zn). Duckweeds have been shown to differ in their tolerances to Zn; however, despite them most commonly co‐occurring with other species, there is a lack of research concerning the effect of species interactions on Zn tolerance. Here, we tested whether the presence of a second species influenced the growth rate of the three duckweed species *Lemna minor*, *Lemna gibba*, and *Lemna turionifera*. We used four different Zn concentrations in a replicated microcosm experiment under sterile conditions, either growing the species in isolation or in a two‐species mixture. The response to Zn differed between species, but all three species showed a high tolerance to Zn, with low levels of Zn even increasing the growth rates. The growth rates of the individual species were influenced by the identity of the competing species, but this was independent of the Zn concentration. Our results suggest that species interactions should be considered in future research with duckweeds and that several duckweed species have high tolerance to metal pollution, making them candidates for phytoremediation efforts.

## Graphical abstract

We tested whether the presence of a second species influenced the growth rate of the three duckweed species *Lemna minor*, *Lemna gibba*, and *Lemna turionifera*. We used four different Zn concentrations in a replicated microcosm experiment, either growing the species in isolation or in a two‐species mixture. The response to Zn differed between species, but all three species showed a high tolerance to Zn. The growth rates of the individual species were influenced by the identity of the competing species, but this was independent of the Zn concentration.

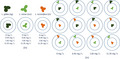

## 1 INTRODUCTION

Duckweeds are small, floating aquatic plants with a simplistic morphology. Individual duckweeds consist of single fronds with zero to multiple roots attached to the bottom surface. Duckweeds can flower; however, they rarely do so (Hicks, 1932). Their main reproduction strategy consists of asexual budding, where several daughter fronds bud and then detach from the mother frond (Laird & Barks, 2018). An individual frond can produce up to a couple dozen of daughter fronds over its life, which is only a few weeks short. Duckweeds are found around the world in slow‐flowing freshwater systems, when suitable anchoring possibilities are present (Landolt, 1986). Their fast reproduction cycle and their short life span make duckweeds useful model organisms for research in ecology and evolution (Laird & Barks, 2018). Duckweeds can tolerate high levels of nitrogen, phosphorus, and heavy metals, and different species can have different responses to temperature, light, nutrients, and toxicants (Landolt, 1996). In nature, duckweed species frequently co‐exist (Landolt, 1986).

Zn is an essential trace element for plant growth, but elevated concentrations inhibit growth and can lead to chlorosis. Therefore, elevated Zn levels are phytotoxic (Rout & Das, 2009). Zn is a commonly used building material and through run‐off from roofs, galvanized items, and pipes it finds its way into waters and sediments, leading to Zn pollution in urbanized areas (AWEL, 2006). Between 2006 and 2014, Zn concentration measurements exceeded the indicator value of 5 µg/L at 15 groundwater measuring stations across Switzerland, more than any other trace element measured (Bundesamt für Umwelt BAFU, 2019).

In the face of pollution of water systems, *Lemnaceae* are studied as potential organisms for phytoremediation (Liu et al., 2021). For example, one species of duckweed, *Lemna minor* L. (common duckweed), has shown to be a good accumulator of heavy metals such as cadmium, selenium, and copper (Zayed et al., 1998). Several studies have shown metal accumulation in different duckweed species (Lahive et al., 2011), which depended both on the species (Cardwell et al., 2002) and on the metal (Gaur et al., 1994). A prerequisite for metal accumulation, however, is the tolerance of a species to elevated levels of heavy metals. Duckweed species differ in their tolerance to Zn: *L*. *minor* was shown to tolerate Zn concentrations above 100 mg/L but the gibbous duckweed *Lemna gibba* only tolerated concentrations up to 10 mg/L (Lahive et al., 2011). Zn tolerance of other duckweed species such as *Lemna turionifera* (red duckweed) was, to our knowledge, never investigated.

Additionally, there is a lack of research concerning the influence of species interactions on duckweed resistance to metal pollution. Previous studies suggest that species interactions in duckweeds can influence growth rates (Clatworthy & Harper, 1962; Gopal & Goel, 1993; Peeters et al., 2016). Here, we hypothesized that the presence of a second species in a mixed setting could increase heavy metal tolerance because of facilitation. The stress‐gradient hypothesis predicts that interactions among plants are context‐dependent, shifting from competition to facilitation as environmental stress increases (Callaway, 1995). At high Zn concentrations, if the more tolerant duckweed species accumulate Zn present in the medium, this could facilitate the persistence or even growth of the co‐occurring, less heavy‐metal tolerant species. However, at low levels of Zn concentration, competition for nutrients could override any facilitative mechanisms, leading to a negative effect of the presence of a second species.

To test our hypothesis, we grew three *Lemnaceae* species in isolation and in two‐species pairings along a zinc sulfate (ZnSO_4_) concentration gradient (0, 0.45, 1.82, 11.35 mg/L Zn). We chose to use a gradient that is relevant in terms of Zn pollution in Europe (1.3 mg/L on average, Zhou et al., 2020). The lowest concentration used was below the European average, the second lowest just above but below the indicator level (5 mg/L), and finally, the highest concentration level exceeded that found in a waterbody near a mining area in Turkey (7.23 mg/L, Sasmaz et al., 2015). Thus, the highest concentration used would represent a heavily polluted waterbody, where phytoremediation could be applied. We measured the Zn tolerance of three duckweeds species *L*. *minor*, *L. gibba*, and *L*. *turionifera* over 17 days in replicated microcosms under sterile and controlled conditions.

## 2 MATERIALS AND METHODS

### 2.1 Duckweed cultures

Axenic cultures of the three duckweed species were maintained at the Department of Evolutionary Biology and Environmental Studies, University of Zürich. Three strains were delivered in July from the Landolt Duckweed Collection (www.duckweed.ch): *Lemna turionifera*, strain 9478, sourced from Racibórz, Silesian, Poland, *Lemna minor*, strain 9978, sourced from Oberegg, Appenzell Innerrhoden, Switzerland, and *Lemna gibba*, strain 9965, sourced from Schloss Wartensee, Rorschacherberg, St. Gallen, Switzerland. The axenic cultures were held on Hoagland's E Medium (see Appendix Table S2 for the recipe used) in incubators at 18°C with a light regime of 14/10 h light/dark.

### 2.2 Experimental set up

The three species occupy a similar ecological niche. They float on the water surface and are very similar in terms of their morphology. In particular, non‐gibbous *L*. *gibba* and *L*. *minor* fronds cannot be distinguished by eye (De Lange & Pieterse, 1973) but the differentiation between *L*. *minor* and *L*. *turionifera* can be equally challenging (Senevirathna et al., 2021). Therefore, we separated the species using a floating ring (Figure S4). Thus, we could overcome previous limitations (Clatworthy & Harper, 1962) and investigate the competition between these closely related species.

For each treatment and the control, 18,250ml‐bottles were filled with 100 ml of Hoagland's E Medium. To create the Zn pollution gradient, ZnSO_4_·7H_2_O (Alfa Aesar, Thermofisher) was added (2, 8, 50 mg/L). Elemental Zn accounts for 65.38 g/mol (22.7%) of the ZnSO_4_·7H_2_O compound, the final concentration of the metal Zn was consequently 0.45 mg/L Zn, 1.82 mg/L Zn, and 11.35 mg/L Zn. The medium was autoclaved before the experiment.

**FIGURE 1:**
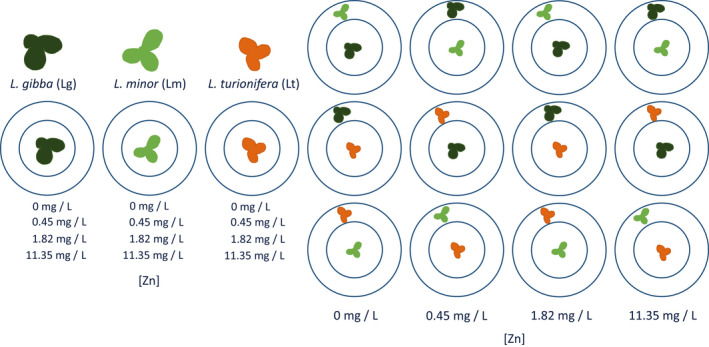
Experimental design with the three different species used. Left: schematic of species growing in the isolated setting (12 treatments replicated three times for a total of 36 bottles). Right: Schematic of species growing in the presence of a competitor (mixed setting). Every treatment *x* species combination was replicated three times for a total of 36 experimental units. The whole experiment had consequently 72 bottles

There were six different compositions (three isolated, three mixed), four Zn concentration levels, and every treatment was replicated three times, thus in total, there were 72 bottles. At the beginning of the experiment, we added 5–7 fronds to the 250 ml bottles according to the study design (Figure 1). In the isolated setting, the 5–7 fronds were added to the inner area (inside the plastic ring). No fronds were added to the outer area. In the mixed setting, 5–7 fronds from one species were added to the inner area and 5–7 fronds from a second species were added to the outer area. We could thus individually track the populations that were either growing alone in a culture bottle or with a population of a competing species (*n* = 108). The inside/outside position of the two respective species was kept for the three replicas but changed depending on the concentration and the specific species composition. So was the position the same for 0 mg/L Zn and 1.82 mg/L Zn, and for 0.45 mg/L Zn and 11.35 mg/L Zn but this differed between the two mixed settings (Figure 1). The bottles were kept in an incubator (Appendix Figure S4) at 20°C with a light regime of 16/8h light/dark for 17 days. During the experiment, the species competed for the same nutrients in an uncrowded culture, but there was no competition for light.

The number of fronds was counted eight times over the course of 17 days. Completely white (dead) fronds were not included in the total number of fronds. During the experiment, the species got mixed in a small number of bottles. Due to their morphological similarity, the species could not be reliably distinguished anymore and consequently any data from these bottles post the mixing event were excluded.

### 2.3 Data analysis

Four populations were excluded from all analyses because they declined rapidly and went extinct despite growing in the medium without the addition of ZnSO_4_. The excluded populations had high leverage on the results but were qualitatively very different from all other populations. The four populations were each a replicate of the pairing *L*. *gibba* and *L*. *minor* at 0 mg/L Zn (two populations), of *L*. *turionifera* in the pairing *L*. *turionifera* and *L*. *minor* at 0 mg/L Zn (one population), and of *L*. *minor* in the isolated setting at 0 mg /L Zn (one population). Subsequently, population growth rates for each individual population (*n* = 104) were calculated as ln(*N*_2_/*N*_1_)/(*t*_1_ − *t*_2_). We used the initial population abundance at the start of the experiment (*t*_1_, 20.07.2021) and the final time point after 17 days (*t*_2_, 6.8.2021) to calculate total growth rates. In the mixed setting, we calculated growth rates for each species separately. Because most other studies examining the influence of Zn on duckweed growth were conducted over less than 10 days, we additionally calculated initial growth rate comparing the first day of the experiment (*t*_1_) with day 8 (*t*_2_, 28.7.2021). Initial growth rates represent growth rates for when we can be confident that nutrients were not limiting but nutrients were likely not limiting throughout the experiment due to the combination of small populations sizes and the nutrient‐rich medium used in the experiment.

Linear models with abundance over time and total growth rate as response terms and position as explanatory variable showed that there was, on average, no significant effect of position (inside vs. outside area, Appendix Figure S1). Due to the study design (Figure 1), position was confounded with the Zn × composition interaction; however, this interaction was never significant (see Appendix Table S1).

Total and initial growth rates were then used as response variables in the linear models. We did analyses of variances (ANOVAS) for each species separately but also the full linear models (see Appendix). The treatment variables were species identity (*L*. *gibba* (Lg), *L*. *minor* (Lm), or *L*. *turionifera* (Lt)), Zn concentration (0, 0.45, 1.82, and 11.35 mg/L Zn), the setting (isolated vs. mixed) and composition (isolated, pairing with species 1, pairing with species 2). lmerTest (Bates et al., 2015; Kuznetsova et al., 2017) was used for the mixed models. Composition and position were included as random factors where appropriate. All analyses were done in R (R version 4.1.0, R Development Core Team, 2021).

## 3 RESULTS

**FIGURE 2:**
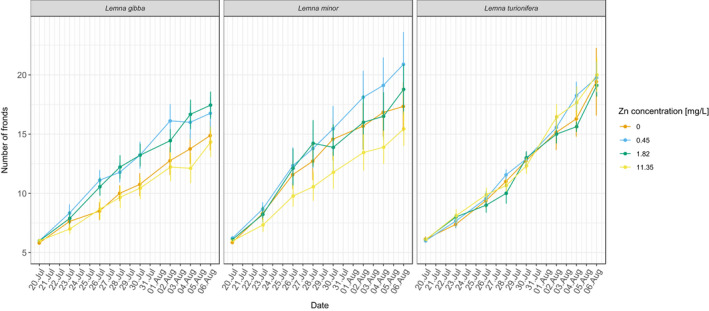
Growth of the three duckweed species in the four zinc (Zn) treatments over 17 days. Shown are means and standard errors for each sampling date, across settings and compositions. Orange lines, 0 mg/L Zn, blue lines, 0.45 mg/L Zn, green lines, 1.82 mg/L Zn, yellow lines, 11.35 mg/L Zn

**FIGURE 3:**
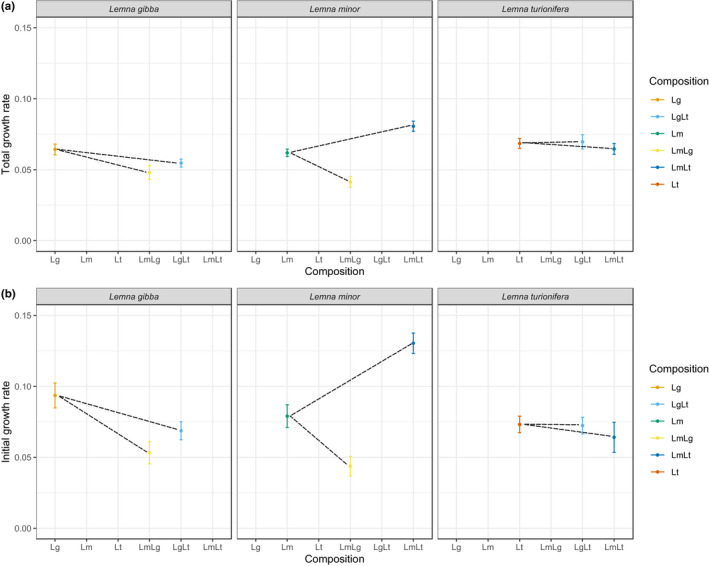
Growth rates across the entire time series (total growth rate, a) and growth rates during the first 8 days of the experiment (initial growth rates, b) of three species separately and in the two settings across the Zn gradient. Orange: isolated setting, blue: mixed setting. Shown are means and associated standard errors across replicates and, in the mixed setting, across the two pairings

Over all concentrations and compositions, we found that *L*. *turionifera* had the highest total growth rate, followed by *L*. *minor* and *L*. *gibba* (Appendix Figures S2, Appendix Figures S3). However, initial growth rate was greatest for *L*. *minor* (Appendix Figures S2, Appendix Figures S3). All three species showed an overall high tolerance to Zn (Figure 2). *L*. *turionifera* was not influenced by Zn pollution. In contrast, the addition of ZnSO_4_ to the experimental cultures significantly influenced *L*. *gibba* and *L*. *minor* but only at the beginning of the experiment (Table 1; Figure 3). *L*. *gibba* profited from intermediate levels of ZnSO_4_ (Figure 3). Similarly, *L*. *minor* grew best at the second‐highest level of Zn concentration (1.82 mg /L), but in contrast to *L*. *gibba*, the second‐lowest level (0.45 mg/L) did not increase growth (Figure 3).

**TABLE 1:** Summary of the Type 3 ANOVAs showing the influence of the setting (isolated vs. mixed), the Zn concentration (factorial), and the composition (isolated, pairing with species 1, pairing with species 2) on the three study species

Species	Total growth rate			Initial growth rate		
Zn concentration	numDF, dDF	*F*	*p*	numDF, dDF	*F*	*p*
*Lemna gibba*	3, 28	1.700	.190	3, 29	3.227	.037
*Lemna minor*	3, 27	2.742	.063	3, 28	3.660	.024
*Lemna turionifera*	3, 29	0.073	.974	3, 31	1.454	.246

Setting (mix vs. Mono)		*F*	*p*		*F*	*p*
*Lemna gibba*	1, 31	5.096	.040	1, 33	11.394	.002
*Lemna minor*	1, 14.48	0.017	.898	1, 23.65	0.377	.544
*Lemna turionifera*	1, 31	0.065	.800	1, 32	0.017	.897

Composition		*F*	*p*		*F*	*p*
*Lemna gibba*	2, 21.62	3.291	.057	2, 31	6.842	.003
*Lemna minor*	2, 30	34.052	.000	2, 32	35.594	.000
*Lemna turionifera*	2, 30	0.371	.693	2, 27.81	0.334	.719

Significant (<.05) and near‐significant (<.06) *p*‐values are in bold. The linear mixed models for setting and composition included position (outer vs. inner) as random factor, the linear mixed model for concentration included composition as random factor. Within species, interaction terms were never significant and thus not shown here (but see Appendix Table S1). numDF: numerator degrees of freedom, dDF: denominator degrees of freedom.

**FIGURE 4:**
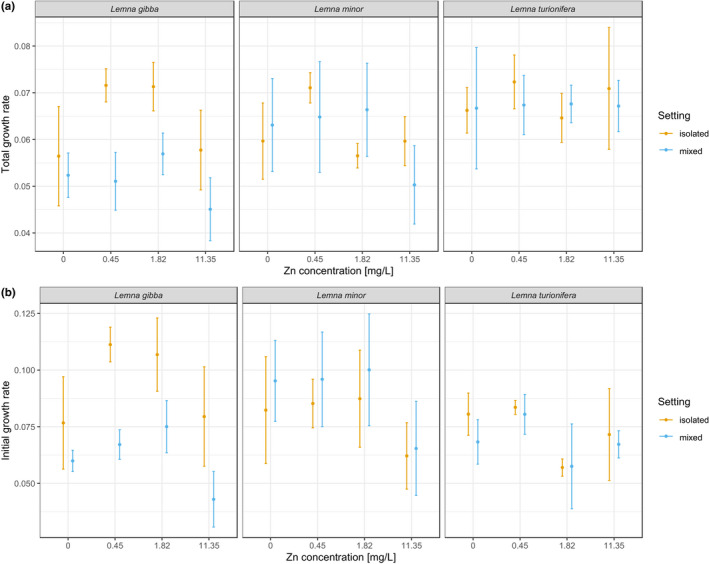
(a) Growth rates across the entire time series and (b) for the first 8 days of the experiment (initial growth rates) of the three species for each composition separately. Means and associated standard errors across all Zn treatments (including no Zn) are presented. For initial growth rate, the interaction between composition and concentration was not significant, but for total growth rate it was (Appendix Table S1). Dashed lines are drawn to visualize the difference between the isolated setting and the two different pairings

The setting (isolated vs. mixed) only significantly influenced *L*. *gibba* growth rates (Table 1), specifically, *L*. *gibba* profited from growing alone and showed significantly lower growth rates (initial and total) when paired with either *L*. *minor* or *L*. *turionifera* (also mirrored in significant effects of composition, Table 1). For *L*. *minor* it depended on the pairing (significant effects of composition on the individual species’ growth rate, Table 1; Figures 3, 4). *L*. *minor* performed better when paired with *L*. *turionifera* and worse when paired with *L*. *gibba*. *L*. *turionifera* showed no difference in growth rate when paired with either *L*. *gibba* or *L*. *minor* (Table 1; Figure 4). The positive and negative effects of the three species on each other are summarized in Table 2.

**TABLE 2:** Overview facilitative (+) and inhibiting (−) effects between the species in the experiment (effect of species on the left on the species on the right)

	on *L*. *gibba*	on *L*. *minor*	on *L*. *turionifera*
Total growth rates			
Effect of *L*. *gibba*		–*	no effect
Effect of *L*. *minor*	–*		– ns
Effect of *L*. *turionifera*	–ns	+**	
Initial growth rates			
Effect of *L*. *gibba*		–^a^	no effect
Effect of *L*. *minor*	–***		– ns
Effect of *L*. *turionifera*	–*	+***	

Note

Significant effects are labelled with ^a^ *p* < .1, *p < .05, **p < .01, ***p < .001. See also Figure 4.

## 4 DISCUSSION

### 4.1 Zn tolerance was high in all three studied species

Contrary to expectations, growth rates were not highest in the treatments without the addition of Zn. Low levels of Zn even increased growth rates for two of the species studied (*L*. *gibba* and *L*. *minor*). Zn is an essential plant nutrient, and Zn deficiency has been shown to reduce fresh weight production in *L*. *gibba* (Vaughan et al., 1982). Zn is present in the Hoagland's E Medium; nevertheless, it is possible that the *Lemna* species are Zn‐limited in this medium and, therefore, show a lower growth rate as a sign of Zn deficiency. All three *Lemnaceae* species showed a very high tolerance to Zn. Only *L*. *minor* exhibited reduced growth rates in the highly polluted environment (11.35 mg/L). The high Zn tolerance for *L*. *gibba* contrasts previous work showing that concentrations of 4 mg/L of Zn inhibited growth by 50% (Lahive, O’ Halloran, et al., 2011; Megateli et al., 2009) and 10 mg/L of Zn reduced specific biomass growth by 90% (Lahive, O’ Halloran, et al., 2011).

For *L*. *minor*, the results partially confirm previous work. Jayasri and Suthindhiran (2017) showed that *L*. *minor* increased biomass yield in fronds treated with a lower concentration (0.5 mg/L) of Zn by 30% compared to the control. However, in their 4‐day experiment 10 mg/L had little effect on growth (Jayasri & Suthindhiran, 2017). Only 20 mg/L started inhibiting growth of *L*. *minor*. This contrasts our results that found that 11.35 mg/L reduced growth rates of *L*. *minor* (significantly so in comparison with 1.82 mg/L). However, our findings also suggest that at lower concentrations Zn promotes the growth of *L*. *minor*, but inhibits its growth at concentrations higher than 10 mg/L. Lahive, O’Callaghan, et al. (2011) reported that *L*. *minor* tolerated Zn concentration above 100 mg/L, but specific biomass growth rate was reduced significantly at low concentrations of 3 mg/L (reduction by 20%). Whether some of these discrepancies could also have been due to different culture mediums used (e.g., Hoagland's medium used in our experiment vs. Hutners medium used in Lahive, O’Callaghan, et al. (2011) vs. Coic and Lessaint solution used in Jayasri & Suthindhiran (2017)) remains untested.

The duration of our experiment exceeded that of previous experiments, which were conducted over 4–7 days (e.g., Jayasri & Suthindhiran, 2017; Lahive, O’ Halloran, et al., 2011; Megateli et al., 2009). We speculated that these previous studies may have underestimated the toxicity of Zn (and overestimated the duckweed Zn tolerance) because the plants may become more sensitive over time, as their tissue accumulates more Zn. However, the overall high tolerance to Zn we found after 17 days suggests that toxicity does not increase over time. The high tolerance of duckweed to polluted environments might be an explanation as to why duckweeds are common all around the world (Landolt, 1986).

### 4.2 Mixing two species can have contrasting effects on the focal species

Based on the stress‐gradient hypothesis (e.g., Callaway et al., 2002), we hypothesized that in a more stressful environment we would observe more facilitative interactions while in the medium without Zn pollution we would observe more competitive interactions. We did not find evidence for this hypothesis, since the levels of Zn pollution we used in our experiment were not high enough to impact duckweed growth rates. Instead, we found that species interactions were highly dependent on focal species identity and competitor identity. For example, the effect of the setting on growth rate was only consistent for one of the three species, *L*. *gibba*. However, contrary to expectations, it did not profit from growing in the presence of a second species that could have helped it to accumulate Zn. Instead, *L*. *gibba* grew the best alone. Previous research with *L*. *gibba* competing against a different duckweed species, the greater duckweed *Spirodela polyrhiza*, showed that *L*. *gibba* was highly competitive due to its gibbous fronds which could overgrow *S*. *polyrhiza* (Clatworthy & Harper, 1962). However, in our experiment there was no physical contact between the species and *L*. *gibba* only produced non‐gibbous fronds. The outcome of species pairings could, therefore, be dependent on frond morphology, which in turn is dependent on the environmental conditions. Gibbosity in *L*. *gibba* depends on the environmental conditions, and may be restricted to optimal growth conditions, including very high nutrient availability (Vaughan & Baker, 1994). Under our conditions in the experiment, *L*. *gibba* may have had a disadvantage due to the lack of gibbous fronds and the inability to overgrow the competitor.

For the other two species, growth performance depended on the pairing, but there was no evidence of facilitation. Instead, we observed strong competition between *L*. *minor* and *L*. *turionifera*, with *L*. *minor* outcompeting *L*. *turionifera*. To the best of our knowledge, this is the first study investigating *L*. *turionifera* growth rates in the presence or absence of a competitor. Recently, it has been found that some populations previously thought to be *L*. *minor* were in fact *L*. *turionifera* (Senevirathna et al., 2021). It is thus possible that *L*. *turionifera* has a wider geographic distribution than previously thought and is, therefore, a good candidate for phytoremediation in many regions of the world.

For *L*. *minor*, we found that, even though it profited from growing with *L*. *turionifera*, the presence of *L*. *gibba* had a negative impact on its growth. Simultaneously, *L*. *gibba* was negatively influenced by the presence of *L*. *minor*. Thus, this pairing had a negative effect on both interacting species, which is surprising given that they frequently co‐exist in nature. Due to their morphological similarity, *L*. *gibba* and *L*. *minor* have rarely been used in competition experiments. In instances where they did compete in experiments the analysis was not completed (Clatworthy & Harper, 1962; Wołek, 1972), or the two species were even grouped together (Peeters et al., 2016). As an exception, Rejmánková (1975) found that in gibbous form, *L*. *gibba* was always the stronger competitor, overgrowing *L*. *minor* (Rejmánková, 1975).

Duckweeds have great potential for heavy metal removal in wastewaters (Abdel‐Gawad et al., 2020) but many questions remain, in particular in terms of how polycultures of multiple species may improve efficiency. Our study is a first assessment of the interaction between competition and tolerance to Zn pollution for a subset of *Lemnaceae* species. We do acknowledge that there might be large variation among ecotypes (genotypes) from the same species (Ziegler et al., 2015). Thus, a next step should be to test the accumulation of Zn not only for species in an isolated setting across different metal concentrations (Lahive, O’Callaghan, et al., 2011) but also in a mixed setting and, importantly, in a natural environment with duckweed populations representing also natural genetic variation. In addition, due to their fast growth, it is possible that duckweed species will evolve in response to their competitor, especially when there is intraspecific genetic diversity present (Hart et al., 2019). For future phytoremediation efforts, species mixing could be interesting, but the effects of a second species need to be evaluated first as they cannot be readily predicted from the performance in individual cultures.

## CONFLICT OF INTEREST

The authors declare no conflict of interest.

## AUTHOR CONTRIBUTION

**Lorena Lanthemann:** Conceptualization (equal); Data curation (equal); Formal analysis (supporting); Investigation (equal); Methodology (equal); Project administration (supporting); Visualization (supporting); Writing – original draft (equal); Writing – review & editing (supporting). **Sofia J. van Moorsel:** Conceptualization (equal); Data curation (equal); Formal analysis (lead); Funding acquisition (lead); Investigation (lead); Methodology (equal); Project administration (lead); Supervision (lead); Visualization (equal); Writing – original draft (equal); Writing – review & editing (equal).

## Supporting information

Supinfo S1

## Data Availability

Data is available on Data Dryad (https://doi.org/10.5061/dryad.h9w0vt4kj).
