# Supplementary material for: Species interactions in three Lemnaceae species growing along a gradient of zinc pollution
Source: Ecol Evol. 2022 Feb 18;12(2):e8646. doi: 10.1002/ece3.8646 (PMC8855331; doi:10.1002/ece3.8646)
Supplement: Supplementary file 1 — Supinfo S1 [file ECE3-12-e8646-s001.docx]

**APPENDIX**

**Appendix Table S1. ANOVA table for linear model.** Top: Initial growth rate as response variable. Bottom: Total growth rate as response variable. Significant p-values (< 0.05) are highlighted in bold.

| **Response: Initial growth rate** |  |  |  |  |  |
| --- | --- | --- | --- | --- | --- |
| **Source of variation** | **Df** | **Sum Sq** | **Mean Sq** | ***F*** | ***P*** |
| Concentration | 3 | 0.007421 | 0.0024738 | 4.374 | **0.007** |
| Species | 2 | 0.004917 | 0.0024583 | 4.347 | **0.017** |
| Setting | 1 | 0.002044 | 0.0020441 | 3.614 | 0.061 |
| Composition | 4 | 0.039897 | 0.0099742 | 17.635 | **< 0.001** |
| Concentration x Species | 6 | 0.006559 | 0.0010932 | 1.933 | 0.087 |
| Concentration x Setting | 3 | 0.000175 | 0.0000584 | 0.103 | 0.958 |
| Concentration x Composition | 12 | 0.008633 | 0.0007194 | 1.272 | 0.254 |
| Species x Composition | 1 | 0.011968 | 0.0119677 | 21.160 | **< 0.001** |
| Residuals | 71 | 0.040156 | 0.0005656 |  |  |
| **Response: Total growth rate** |  |  |  |  |  |
| **Source of variation** | **Df** | **Sum Sq** | **Mean Sq** | ***F*** | ***P*** |
| Concentration | 3 | 0.0010202 | 0.00034008 | 2.246 | 0.091 |
| Species | 2 | 0.0023376 | 0.00116881 | 7.719 | **0.001** |
| Setting | 1 | 0.0005907 | 0.00059075 | 3.901 | 0.052 |
| Composition | 4 | 0.0070683 | 0.00176709 | 11.670 | **< 0.001** |
| Concentration x Species | 6 | 0.0007715 | 0.00012858 | 0.849 | 0.537 |
| Concentration x Setting | 3 | 0.0004804 | 0.00016013 | 1.058 | 0.373 |
| Concentration x Composition | 12 | 0.0025644 | 0.0002137 | 1.411 | 0.183 |
| Species x Composition | 1 | 0.002356 | 0.00235599 | 15.559 | **< 0.001** |
| Residuals | 67 | 0.0101457 | 0.00015143 |  |  |

**Appendix Table S2. Recipe for Hoagland’s E Medium.** The pH was set to 5.7 (5.71, 5.76, 5.7, 5.75 for the four bottles of 2-L medium) before autoclaving the medium.

| 2 L contained |  |
| --- | --- |
| 5 mL | MgSO_4_ |
| 5 mL | Ca(NO_3_) x 4 H_2_O |
| 5 mL | K_2_HPO_4_ |
| 5 mL | KNO_4_ |
| 5 mL | micronutrients (H_3_BO_3_, MnCl_2_ x 4H_2_O, ZnSO_4_ x 7 H_2_O, NaMoO_4_ x 2 H_2_O, CuSO_4_ x 5 H_2_O) |
| 50 mL | Fe-EDTA (FeCl_3_ x 6 H_2_O, EDTA) |

**Appendix Figure S1. A)** Influence of position (inner vs. outer area of the ring) over time. On some dates, the inner position (red bars) tended to have a positive effect on growth but not significantly so (*F_1,532_*= 0.2025, *P* = 0.6529 for the main term position and *F_3,532_* = 0.1172, *P* = 0.7322 for the interaction term position x date). The isolated setting is not shown here, as only the inner position was used for that treatment. B) Influence of position on total growth rates for each species separately. When *L. gibba* was grown on in the inside of the floating ring, on average, across all compositions and treatments, it tended to grow better. However, the test statistics were not significant (*F_1,59_* = 0.361, *P_2,59_* = 0.56 for the main term position and *F* =0.837, *P* = 0.44 for the interaction term position x species).

**Appendix Figure S2.** Growth of all duckweed populations averaged across the three replicates over the 17 days of the experiment.

**
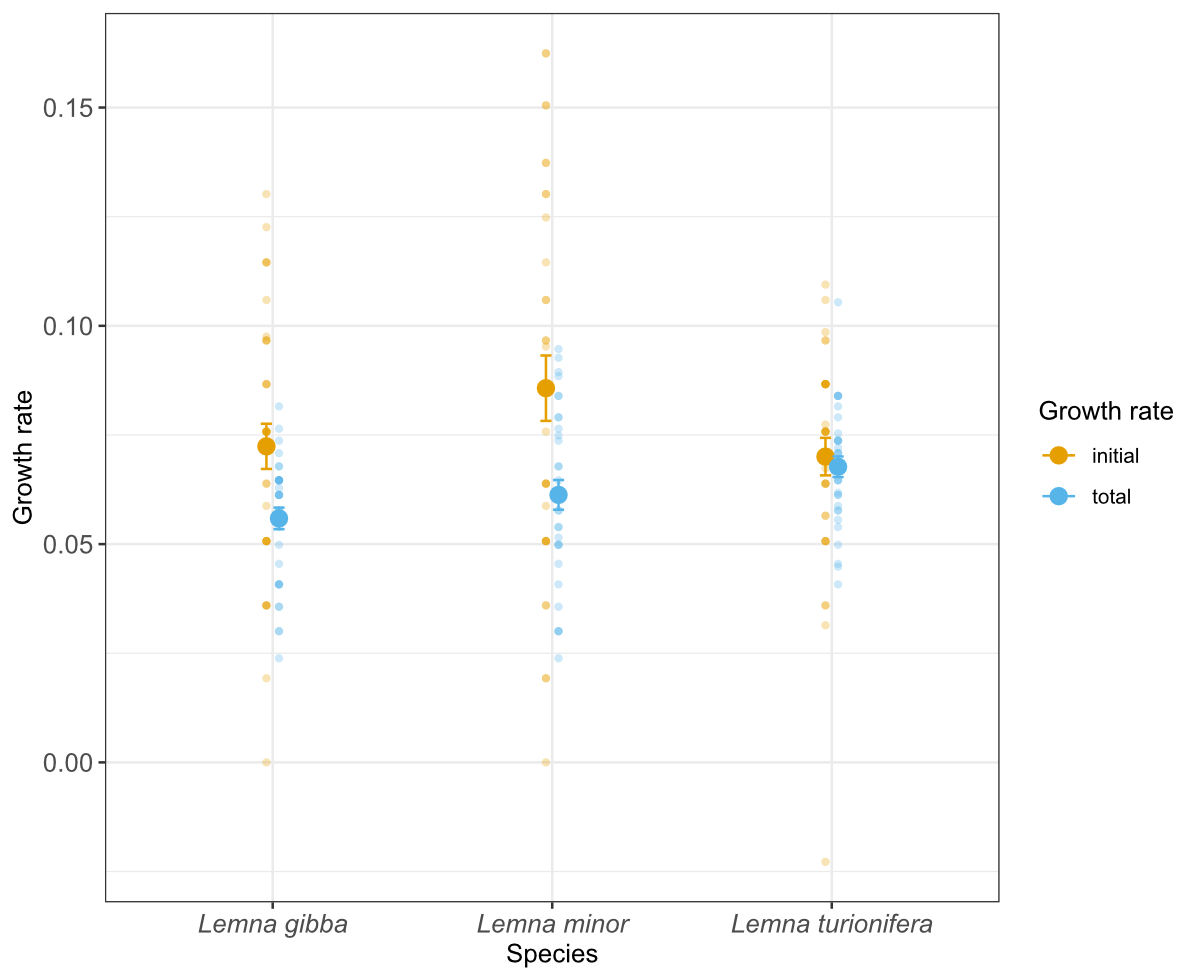
Appendix Figure S3.** Total (blue) and initial (orange) growth rate per species across all compositions and concentrations. Shown are mean and standard errors. All data points are plotted in the background (smaller points).


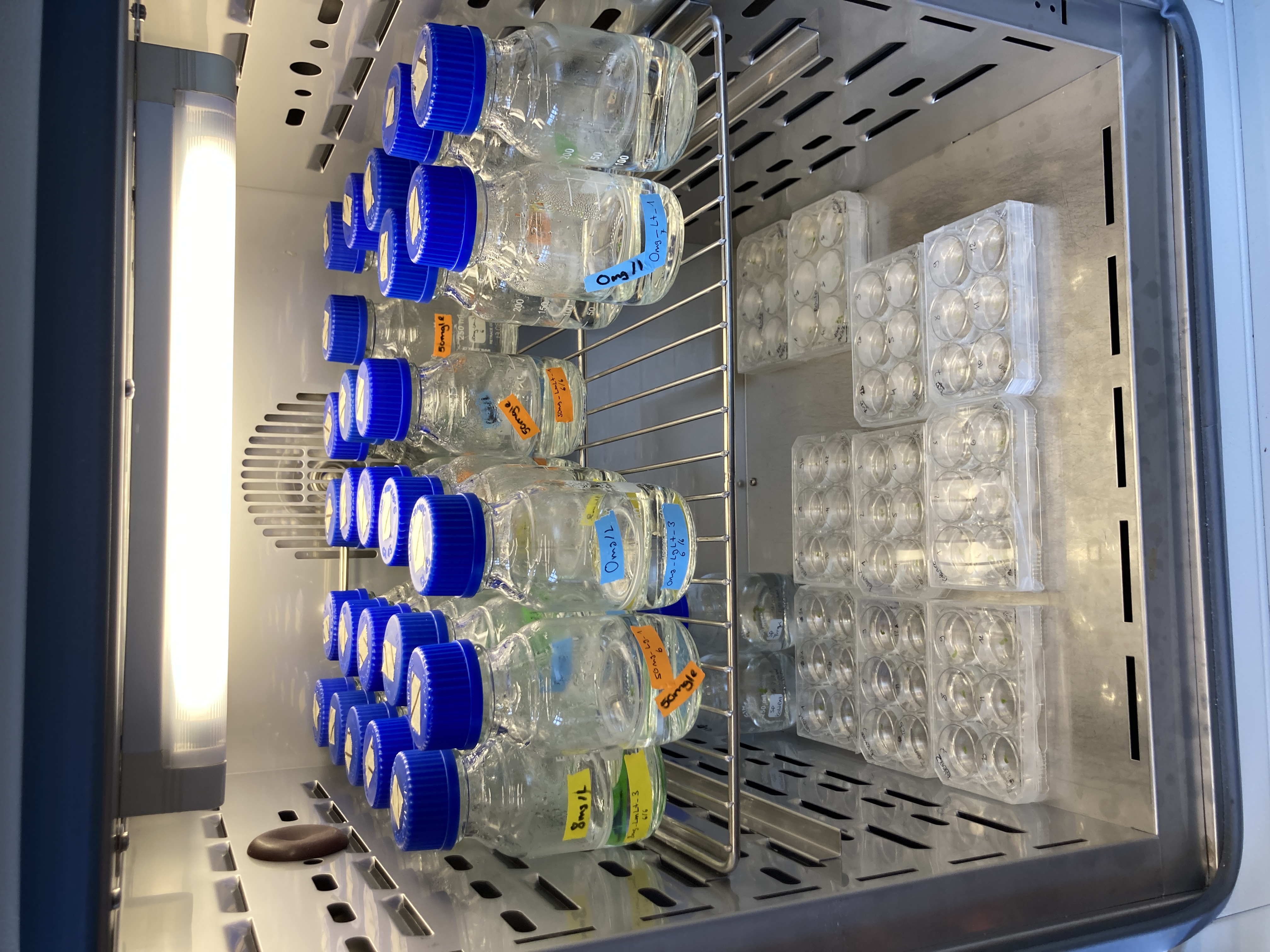

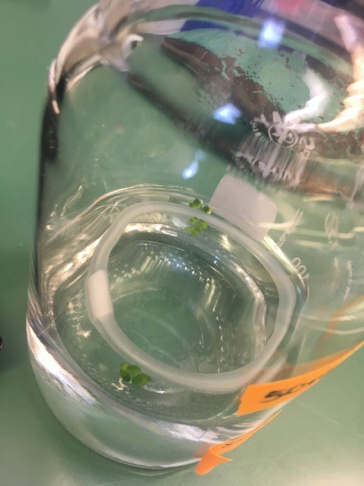


**Appendix Figure S4. Photos of the experimental microcosms.** Left: The plastic ring separating the two species. Right: The different microcosms growing in the incubator.
